# *Pratylenchus smoliki*, a new nematode species (Pratylenchidae: Tylenchomorpha) from the Great Plains region of North America

**DOI:** 10.21307/jofnem-2021-100

**Published:** 2021-12-10

**Authors:** Thomas Powers, Timothy Todd, Tim Harris, Rebecca Higgins, Ann MacGuidwin, Peter Mullin, Mehmet Ozbayrak, Kirsten Powers, Kanan Sakai

**Affiliations:** 1 Department of Plant Pathology, University of Nebraska-Lincoln, Lincoln, NE 68583-0722; 2 Department of Plant Pathology, Kansas State University, Manhattan, KS 66506; 3 Department of Plant Pathology, University of Wisconsin-Madison, Madison WI; 4 Department of Entomology, Bornova Plant Protection Research Institute, 35040 Bornova/Izmir, Turkey; 5 International Institute of Tropical Agriculture, Nairobi 00100, Kenya

**Keywords:** COI, Nematode distribution, Phylogeny, Plant-parasitic nematodes, Root-lesion nematode, Taxonomy

## Abstract

*Pratylenchus smoliki* is a new species of root-lesion nematode described from corn-soybean production fields in the Central Great Plains of North America. It is characterized by populations with relatively abundant males, two lip annuli, females with a round functional spermatheca and a conoid to subcylindrical tail with a non-crenate, smooth terminus. In host preference tests, corn and wheat produce the largest nematode populations, whereas sorghum and soybeans produce less than 20% the numbers observed on corn. Scanning electron microscopy reveals that the *en face* patterns compare to those seen in *Pratylenchus pseudocoffeae*, *P*. *scribneri, P. hexincisus*, and *P. alleni.* The pattern is described as rectangular to trapezoidal subdorsal and subventral lips adjoining oral disc, but with a clear demarcation between the oral disc and the subdorsal and subventral sectors. A Maximum Likelihood COI tree recognizes *P*. *smoliki* as a moderately-well-supported clade with several haplotype subgroups. A Maximum Likelihood partial 28S tree provides strong support for the *P. smoliki* clade and reinforces the close relationships between species with similar *en face* patterns. Topotype specimens of *P*. *alleni* were demonstrably different from *P. smoliki* using DNA markers. The geographic range of *P. smoliki* overlaps with the ranges of *P. alleni*, *P. scribneri*, *P. neglectus*, *P*. *hexicisus*, and *P. dakotaensis*. The observed host range (corn, rye, sunflower, and wheat) suggests that *P. smoliki* may be native to the tallgrass prairie region of the Great Plains.

Globally, there are 103 described species of *Pratylenchus* according to a recent accounting (Nguyen et al., 2019). A regional survey of the Central Great Plains of North America, conducted using a DNA barcoding approach, recorded nine described species and a roughly equivalent number of potentially undescribed species (Ozbayrak et al., 2019). One of those undescribed species, previously referred to as *Pratylenchus* sp. 1 in Ozbayrak et al. (2019) was described as *Pratylenchus dakotaensis* (Handoo et al., 2021). In this manuscript, we describe a second of those undescribed species, previously referred to as *Pratylenchus* sp. 2 in Ozbayrak et al. (2019), herein described as *Pratylenchus smoliki* n. sp. The new species has been collected from two Great Plains States: Kansas and Nebraska. All positive collections were obtained from fields in a corn-soybean rotation. Notably *P. smoliki* n. sp. reproduces sexually and males make up approximately 30% of the adult population. The possibility that the species identity might be *Pratylenchus alleni* (Ferris, 1961), another two-lipped species with males, led to the collection of that species at its type locality in Saline County, Illinois. *Pratylenchus alleni* has been given a pest rating of “A” by the California Department of Agriculture, suggesting that the species is an organism of known economic importance and subject to action enforced by the state (http://blogs.cdfa.ca.gov/Section3162/?p=1955). Therefore, the comparison of *P. alleni* and *P*. *smoliki* n. sp. has economic as well as scientific relevance.

DNA barcoding with the COI mitochondrial gene has been an accurate approach for species identification in *Pratylenchus* (Troccoli et al., 2016; Singh et al., 2018; Nguyen et al., 2019; Ozbayrak et al., 2019; Handoo et al., 2021). Since COI provides differentiation at the population level as well as species-level discrimination, it is necessary to generate an estimate of within-species genetic variation for the mitochondrial marker. The determination of species relationships, however, requires additional genetic markers, an examination of morphological characters, and an understanding of ecological and physiological characteristics. Presently, empirical evidence supports a strong correspondence between COI haplotype groups and described species boundaries in *Pratylenchus*. In this study, we have constructed a maximum likelihood tree of COI that includes *P. smoliki* n. sp., 22 other described species that are represented by a minimum of two specimens, and nine other described *Pratylenchus* species that are represented by a single specimen. Sequence characterization by 28S and ITS1 is also presented. Species delimitation and host associations for the major *Pratylenchus* species of the Central Great Plains were included in Ozbayrak et al. (2019). Mean genetic distances between species and within species COI diversity have been recalculated to incorporate new specimens. The further characterization of *Pratylenchus smoliki* n. sp. from that publication and the formal description of this species is the primary objective of this study.

## Materials and methods

### Nematode populations

Nematodes included in this study were collected as part of a series of plant disease surveys as described in Ozbayrak et al. (2019). *Pratylenchus smoliki* n. sp. was collected from Buffalo County, Nebraska and Shawnee County, Kansas as part of a wheat and corn soil survey. Comparative host tests and most morphological analyses were conducted on specimens cultured at Kansas State University, originally collected from the type locality. The original isolation of *P. smoliki* n. sp. was from corn root samples collected from experimental seed treatment trials at the Kansas River Valley Experiment Field located 3.5 miles east of Silver Lake, Kansas, U.S.A (Shawnee County). Nematode populations were subsequently maintained at the Kansas State University Throckmorton greenhouse complex in Eudora silt loam planted with the corn hybrid DKC60-69RIB. Nematodes for morphological and molecular analyses were extracted from root incubations as described by Georgi et al. (1983) and shipped as living nematodes in tap water to the Powers lab at the University of Nebraska-Lincoln. Sample collection sites (county locations), nematode identification (NID) numbers, and host information for each analyzed specimen are presented in Table 1.

**Table 1.** *Pratylenchus* specimens used for phylogenetic analysis in this study.

NID #	Species	Stage	Locality	Host	Marker	GenBank#
N3873	*P. smoliki* n. sp.	M	Shawnee Co., KS^*^	Corn	COI	MK878313
N3873	*P. smoliki* n. sp.	M	Shawnee Co., KS^*^	Corn	rDNA LSU	OK490313
N3880	*P. smoliki* n. sp.	M	Shawnee Co., KS^*^	Corn	COI	MK878314
N3880	*P. smoliki* n. sp.	M	Shawnee Co., KS^*^	Corn	ITS1	OK490336
N8136	*P. smoliki* n. sp.	M	Shawnee Co., KS^*^	Corn	COI	MK878315
N8137	*P. smoliki* n. sp.	F	Shawnee Co., KS^*^	Corn	COI	MK878316
N8139	*P. smoliki* n. sp.	F	Shawnee Co., KS^*^	Corn	COI	MK878317
N8139	*P. smoliki* n. sp.	F	Shawnee Co., KS^*^	Corn	rDNA LSU	OK490314
N8145	*P. smoliki* n. sp.	F	Shawnee Co., KS^*^	Corn	COI	MK878318
N8145	*P. smoliki* n. sp.	F	Shawnee Co., KS^*^	Corn	rDNA LSU	OK490315
N8146	*P. smoliki* n. sp.	F	Shawnee Co., KS^*^	Corn	COI	MK878319
N8148	*P. smoliki* n. sp.	F	Shawnee Co., KS^*^	Corn	COI	MK878320
N8154	*P. smoliki* n. sp.	F	Shawnee Co., KS^*^	Corn	COI	MK878321
N8156	*P. smoliki* n. sp.	F	Shawnee Co., KS^*^	Corn	COI	MK878322
N8170	*P. smoliki* n. sp.	F	Shawnee Co., KS^*^	Corn	COI	MK878323
N8170	*P. smoliki* n. sp.	F	Shawnee Co., KS^*^	Corn	rDNA LSU	OK490316
N9793	*P. smoliki* n. sp.	F	Shawnee Co., KS^*^	Corn	COI	OK489823
N9793	*P. smoliki* n. sp.	F	Shawnee Co., KS^*^	Corn	rDNA LSU	OK490317
N9793	*P. smoliki* n. sp.	F	Shawnee Co., KS^*^	Corn	rDNA SSU	OK490342
N9795	*P. smoliki* n. sp.	M	Shawnee Co., KS^*^	Corn	rDNA SSU	OK490343
N9809	*P. smoliki* n. sp.	F	Shawnee Co., KS^*^	Corn	ITS1	OK490337
N10037	*P. smoliki* n. sp.	F	Buffalo Co., NE	Corn	COI	MK878324
N10039	*P. smoliki* n. sp.	F	Buffalo Co., NE	Corn	COI	MK878325
N10053	*P. smoliki* n. sp.	F	Buffalo Co., NE	Corn	COI	MK878326
N11506	*P. smoliki* n. sp.	F	Shawnee Co., KS^*^	Corn	COI	OK489824
N11506	*P. smoliki* n. sp.	F	Shawnee Co., KS^*^	Corn	rDNA LSU	OK490318
N11507	*P. smoliki* n. sp.	F	Shawnee Co., KS^*^	Corn	COI	OK489825
N11508	*P. smoliki* n. sp.	F	Shawnee Co., KS^*^	Corn	COI	OK489826
N11509	*P. smoliki* n. sp.	F	Shawnee Co., KS^*^	Corn	COI	OK489827
N11509	*P. smoliki* n. sp.	F	Shawnee Co., KS^*^	Corn	rDNA LSU	OK490319
N11510	*P. smoliki* n. sp.	F	Shawnee Co., KS^*^	Corn	COI	OK489828
N11592	*P. smoliki* n. sp.	F	Shawnee Co., KS^*^	Corn	COI	OK489829
N11593	*P. smoliki* n. sp.	F	Shawnee Co., KS^*^	Corn	COI	OK489830
N11593	*P. smoliki* n. sp.	F	Shawnee Co., KS^*^	Corn	rDNA LSU	OK490320
N11594	*P. smoliki* n. sp.	F	Shawnee Co., KS^*^	Corn	rDNA SSU	OK490344
N11595	*P. smoliki* n. sp.	F	Shawnee Co., KS^*^	Corn	COI	OK489831
N11595	*P. smoliki* n. sp.	F	Shawnee Co., KS^*^	Corn	rDNA LSU	OK490321
N11600	*P. smoliki* n. sp.	F	Shawnee Co., KS^*^	Corn	COI	OK489832
N11602	*P. smoliki* n. sp.	F	Shawnee Co., KS^*^	Corn	COI	OK489833
N11603	*P. smoliki* n. sp.	F	Shawnee Co., KS^*^	Corn	rDNA LSU	OK490322
N11605	*P. smoliki* n. sp.	F	Shawnee Co., KS^*^	Corn	COI	OK489834
N11607	*P. smoliki* n. sp.	F	Shawnee Co., KS^*^	Corn	COI	OK489835
N11612	*P. smoliki* n. sp.	F	Shawnee Co., KS^*^	Corn	COI	OK489836
N11613	*P. smoliki* n. sp.	F	Shawnee Co., KS^*^	Corn	COI	OK489837
N11614	*P. smoliki* n. sp.	F	Shawnee Co., KS^*^	Corn	COI	OK489838
N11614	*P. smoliki* n. sp.	F	Shawnee Co., KS^*^	Corn	rDNA LSU	OK490323
N11618	*P. smoliki* n. sp.	F	Shawnee Co., KS^*^	Corn	COI	OK489839
N12448	*P. smoliki* n. sp.	M	Shawnee Co., KS^*^	Corn	COI	OK489840
N12451	*P. smoliki* n. sp.	M	Shawnee Co., KS^*^	Corn	COI	OK489841
N12451	*P. smoliki* n. sp.	M	Shawnee Co., KS^*^	Corn	COI	OK489842
N12453	*P. smoliki* n. sp.	M	Shawnee Co., KS^*^	Corn	COI	OK489843
N12453	*P. smoliki* n. sp.	M	Shawnee Co., KS^*^	Corn	rDNA LSU	OK490324
N12454	*P. smoliki* n. sp.	M	Shawnee Co., KS^*^	Corn	ITS1	OK490338
N12457	*P. smoliki* n. sp.	M	Shawnee Co., KS^*^	Corn	COI	OK489844
N12458	*P. smoliki* n. sp.	M	Shawnee Co., KS^*^	Corn	COI	OK489845
N12459	*P. smoliki* n. sp.	M	Shawnee Co., KS^*^	Corn	COI	OK489846
N12463	*P. smoliki* n. sp.	M	Shawnee Co., KS^*^	Corn	COI	OK489847
N12463	*P. smoliki* n. sp.	M	Shawnee Co., KS^*^	Corn	rDNA LSU	OK490325
N12464	*P. smoliki* n. sp.	M	Shawnee Co., KS^*^	Corn	COI	OK489848
N12465	*P. smoliki* n. sp.	F	Shawnee Co., KS^*^	Corn	COI	OK489849
N12466	*P. smoliki* n. sp.	M	Shawnee Co., KS^*^	Corn	COI	OK489850
N12467	*P. smoliki* n. sp.	F	Shawnee Co., KS^*^	Corn	COI	OK489851
N12468	*P. smoliki* n. sp.	F	Shawnee Co., KS^*^	Corn	COI	OK489852
N12469	*P. smoliki* n. sp.	M	Shawnee Co., KS^*^	Corn	COI	OK489853
N12470	*P. smoliki* n. sp.	F	Shawnee Co., KS^*^	Corn	COI	OK489854
N12470	*P. smoliki* n. sp.	F	Shawnee Co., KS^*^	Corn	rDNA LSU	OK490326
N12471	*P. smoliki* n. sp.	F	Shawnee Co., KS^*^	Corn	COI	OK489855
N3717	*P. alleni*	M	Madison Co., NE	Corn	COI	MK877458
N3717	*P. alleni*	M	Madison Co., NE	Corn	rDNA LSU	OK490293
N7381	*P. alleni*	F	Saline Co., IL^**^	Soybean	COI	MK877459
N7382	*P. alleni*	M	Saline Co., IL^**^	Soybean	COI	MK877460
N10848	*P. alleni*	J	Saline Co., IL^**^	Soybean	rDNA LSU	OK490294
N10850	*P. alleni*	F	Saline Co., IL^**^	Soybean	COI	MK877461
N10850	*P. alleni*	F	Saline Co., IL^**^	Soybean	rDNA LSU	OK490295
N8539	*P. crenatus*	F	County Galway, IRE	Pasture	rDNA LSU	OK490296
N9774	*P. crenatus*	J	Rwanda	-	COI	OK489810
N6350	*P. dakotaensis*	F	Atchison Co., KS	Corn	rDNA LSU	OK490297
N6351	*P. dakotaensis*	J	Atchison Co., KS	Corn	rDNA LSU	OK490298
N6438	*P. hexincisus*	F	Graham Co., KS	Corn	rDNA LSU	OK490299
N7726	*P. hexincisus*	F	Big Horn Co., WY	Drybean	rDNA LSU	OK490300
N10849	*P. hexincisus*	J	Saline Co., IL	Soybean	rDNA LSU	OK490301
N10756	*P. neglectus*	F	Seward Co., KS	Corn	rDNA LSU	OK490302
N11481	*P. neglectus*	F	Gallatin Co., MT	Corn	COI	OK489811
N11483	*P. neglectus*	F	Gallatin Co., MT	Corn	COI	OK489812
N11485	*P. neglectus*	F	Broadwater Co., MT	Corn	COI	OK489813
N11487	*P. neglectus*	F	Broadwater Co., MT	Corn	COI	OK489814
N11489	*P. neglectus*	F	Broadwater Co., MT	Corn	COI	OK489815
N11491	*P. neglectus*	F	Treasure Co., MT	Corn	COI	OK489816
N11499	*P. neglectus*	F	Yellowstone Co., MT	Corn	COI	OK489817
N6260	*P. penetrans*	M	Fairbanks Co., AK	Peony	rDNA LSU	OK490303
N6261	*P. penetrans*	J	Fairbanks Co., AK	Peony	rDNA LSU	OK490304
N7091	*P. penetrans*	F	Fairbanks Co., AK	Peony	rDNA LSU	OK490305
N11414	*P. penetrans*	F	British Columbia, CAN	*Prunus* sp.	COI	OK489818
N11415	*P. penetrans*	F	British Columbia, CAN	*Prunus* sp.	COI	OK489819
N11416	*P. penetrans*	F	British Columbia, CAN	*Prunus* sp.	COI	OK489820
N11417	*P. penetrans*	F	British Columbia, CAN	*Prunus* sp.	COI	OK489821
N11424	*P. penetrans*	F	British Columbia, CAN	*Prunus* sp.	COI	OK489822
N6383	*P. scribneri*	F	Marshall Co., KS	Corn	rDNA LSU	OK490306
N7833	*P. scribneri*	J	Custer Co., NE	Corn	rDNA LSU	OK490307
N7839	*P. scribneri*	J	Custer Co., NE	Corn	rDNA LSU	OK490308
N7845	*P. scribneri*	F	Custer Co., NE	Corn	rDNA LSU	OK490309
N10274	*P. scribneri*	F	Kearney Co., NE	Corn	rDNA LSU	OK490310
N10301	*P. scribneri*	F	Phelps Co., NE	Corn	rDNA LSU	OK490311
N10306	*P. scribneri*	F	Phelps Co., NE	Corn	rDNA LSU	OK490312
P130021	*P. scribneri*	-	Maryland	-	ITS1	OK490333
P156024	*P. scribneri*	F	Florida	-	ITS1	OK490334
P344041	*P. scribneri*	-	-	-	ITS1	OK490335
N7566	*P. thornei*	F	Treasure Co., MT	Corn	rDNA LSU	OK490327
N7567	*P. thornei*	F	Treasure Co., MT	Corn	rDNA LSU	OK490328
N11495	*P. thornei*	F	Yellowstone Co., MT	Corn	COI	OK489856
N11497	*P. thornei*	F	Yellowstone Co., MT	Corn	COI	OK489857
N11422	*P. vulnus*	F	British Columbia, CAN	*Prunus* sp.	COI	OK489858
N10764	*Pratylenchus* sp.	F	Sumner Co., KS	Corn	rDNA LSU	OK490329
N12456	*Pratylenchus* sp.	M	Shawnee Co., KS	Corn	COI	OK489859
N10685	*Pratylenchus* sp.	F	Seward Co., KS	Corn	rDNA LSU	OK490330
N8930	*Pratylenchus* sp.	F	Lincoln Co., AR	Soybean	rDNA LSU	OK490331
N10841	*Pratylenchus* sp.	J	Cross Co., AR	Soybean	rDNA LSU	OK490332
P156016	*Pratylenchus* sp.	F	Lancaster Co., NE	Leadplant	ITS1	OK490339
P156019	*Pratylenchus* sp.	F	Gage Co., NE	Big Bluestem	ITS1	OK490340
P201006	*Pratylenchus* sp.	-	Idaho	-	ITS1	OK490341
N84	*Nacobbus aberrans*	M	Xalatlaco, MEX	-	COI	OK489860
N85	*Nacobbus aberrans*	M	Xalatlaco, MEX	-	COI	OK489861
N194	*Nacobbus aberrans*	J	Sioux Co., NE	Sugarbeet	COI	OK489862
N452	*Nacobbus aberrans*	-	Zacatecas, MEX	Drybean	COI	OK489863
N537	*Nacobbus aberrans*	J	Chacra, ARG	Tomato	COI	OK489864
N658	*Nacobbus aberrans*	J	Lisandro Olmos, ARG	-	COI	OK489865

Note: ^*^Type locality for *Pratylenchus smoliki* n. sp. Kansas River Valley Experiment Field, within the 80 acre Paramore Unit, located 3.5 miles east of Silver Lake on U.S. Highway 24, then 1 mile south of Kiro, and 1.5 miles east on 17th Street. ^**^Type locality for *Pratylenchus alleni*. Soybean field 5 miles north of Eldorado, Illinois. Ferris, V. R. (1961).

### Morphological analysis

Nematodes isolated from soil were first examined using a dissecting stereomicroscope. Individual nematodes were mounted in water on temporary glass slides, measured, and digitally photographed using a Leica DMLB light microscope with differential interference contrast optics and a Leica DC300 video camera. A set of 26 standard measurements were taken on individual specimens allowing for the combined retention of morphological and molecular characters. Temporary slides were dismantled after morphological analysis, the nematode was crushed in 18 μl of sterile water with a micropipette tip and frozen in individual PCR microfuge tubes. Both adult females and males, as well as juveniles, were subjected to morphological and molecular analyses. Voucher specimens were fixed in 4% formaldehyde plus 2% glycerol, and the slow evaporation method was used prior to mounting.

Nematodes were prepared for scanning electron microscopy (SEM) by fixation in 4% glutaraldehyde followed by post-fixation with 2% Osmium Tetroxide, dehydration in a graded series of alcohol to 100% ethyl alcohol, critical point drying, mounting on SEM specimen stubs, and coating with silver. Images were obtained on a Hitachi S4700 Field-Emission scanning electron microscope. Microscopic images of all specimens were stored in an in-house database in the Department of Plant Pathology at University of Nebraska-Lincoln.

### Molecular analysis

Polymerase chain reaction amplifications were performed using three different markers: the cytochrome oxidase subunit I (COI), the internal transcribed spacer I (ITS1), and the large-subunit ribosomal RNA (28S) (Table 2). The PCR reaction mixtures and thermocyler conditions for each marker were as follows.

**Table 2.** Primers.

Marker	Primer set	Amplicon Size (kb)	Primer Sequence (5′→ 3′)	Direction	Reference
COI^a^	PsmoF4	0.42	5′-ATY GCS CCC GCC TTT GG-3’	Forward	This manuscript
COI	JB5		5′-AGC ACC TAA ACT TAA AAC ATA ATG AAA ATG-3′	Reverse	Derycke et al. (2005)
COI	JB3	0.43	5′-TTTTTTGGGCATCCTGAGGTTTAT-3′	Forward	Bowles et al., (1992)
COI	JB5		5′-AGCACCTAAACTTAAAACATAATGAAAATG-3′	Reverse	Derycke et al. (2005)
COI	F1KF	0.95	5′-CCTACTATGATTGGTGGTTTTGGTAATTG-3′	Forward	Kanzaki & Futai (2002)
COI	JB5		5′-AGCACCTAAACTTAAAACATAATGAAAATG-3′	Reverse	Derycke et al. (2005)
COI	F7bPrat	0.78	5′-GGDTGRACWTTHTAYCCNCC-3′	Forward	Ozbayrak et al. (2019)
COI	JB5		5′-AGCACCTAAACTTAAAACATAATGAAAATG-3′	Reverse	Derycke et al. (2005)
ITS1^b^	rDNA2	0.62	5′-TTGATTACGTCCCTGCCCTTT-3′	Forward	Vrain et al. (1992)
ITS1	rDNA1.58Sa		5′-ACGAGCCGAGTGATCCACC-3′	Reverse	Cherry et al. (1997)
rDNA LSU^c^	D2A	0.75	5′-ACAAGTACCGTGAGGGAAAGTTG-3′	Forward	De Ley et al. (1999)
rDNA LSU	D3B		5′-TCGGAAGGAACCAGCTACTA-3	Reverse	De Ley et al. (1999)
rDNA LSU	28s-PratF3	0.48	5′-TTTGCAAGTGGAGTGCGT-3′	Forward	This manuscript
rDNA LSU	28s-PratR1		5′-AATAGTTCACCATCTTTCGGG -3′	Reverse	This manuscript

Note: ^a^Cytochrome oxidase subunit I. ^b^Internal transcribed spacer 1. ^c^rDNA Large Subunit.

For a final PCR reaction mixture volume of 30μl/amplification, 5-10 μl of nematode template were added to each reaction mixture of 1.6 μM final concentration for both forward and reverse primers and a 0.05U/μl final concentration of Sigma 2X JumpStart RED Taq ReadyMix. After loading the thermocycler with the reaction mixtures at a Hotstart (94°C), the thermocycler PCR conditions were: one cycle of initial denaturation at 94°C for 5 min, then 35 cycles of denaturation at 94°C for 30 sec; annealing for 30 sec; and extension at 72°C for 90 sec. Annealing temperatures were 50°C, 55°C, and 48°C for COI, ITS1, and 28S amplifications, respectively. A one-cycle final extension stage at 72°C ran for 5 min before the thermocycler program settled at 24°C.

All PCR reactions were conducted on a Techne Prime Thermal Cycler, 60 × 0.5ml (Bibby Scientific Ltd., Staffordshire, UK). To evaluate amplifications, 3 μl of each PCR product was loaded into 1% Low EEO agarose gels and stained with 1% ethidium bromide. Gels were placed into electrophoresis with 0.5X Tris-Borate-EDTA (TBE) running buffer for 35 min at 155V. UV-visualized gel images were digitally recorded. Successful PCR amplifications were purified with a Gel/PCR DNA Fragment Extraction Kit (IBI Scientific, Dubuque, IA) following the manufacturer’s guidelines. Purified DNA amplicons were sequenced in both directions by the UCDNA Sequencing Facility at the University of California-Davis or ETON BioSciences Inc. Sequences were edited and aligned on CodonCode Aligner Version 9.0 (CodonCode Corp, Centerville, Massachusetts). The nucleotide sequences obtained in this study were deposited into the GenBank database (NCBI) under the accession numbers OK489810-OK489865 (COI) and OK490293-OK490344 (28S, ITS1, and 18S).

### Host range trials

Host trials were conducted in 0.5 l Deepots (Stuewe & Sons Inc.) containing pasteurized Eudora silt loam soil from the type location and inoculated with 1,000 to 1,500 nematodes extracted from field-grown corn roots. Trials consisted of (1) commercial corn hybrids, (2) agronomic crops, with two cultivars each of corn, sorghum, soybean, and wheat, and (3) diverse cover crops with corn as the control. Host status was determined by root incubation of eight-week-old plants followed by nematode extraction as described by Georgi et al. (1983). All trials were conducted as randomized complete block designs with four to five replications, and repeated in time once or twice.

## Results

**Figure 1:**
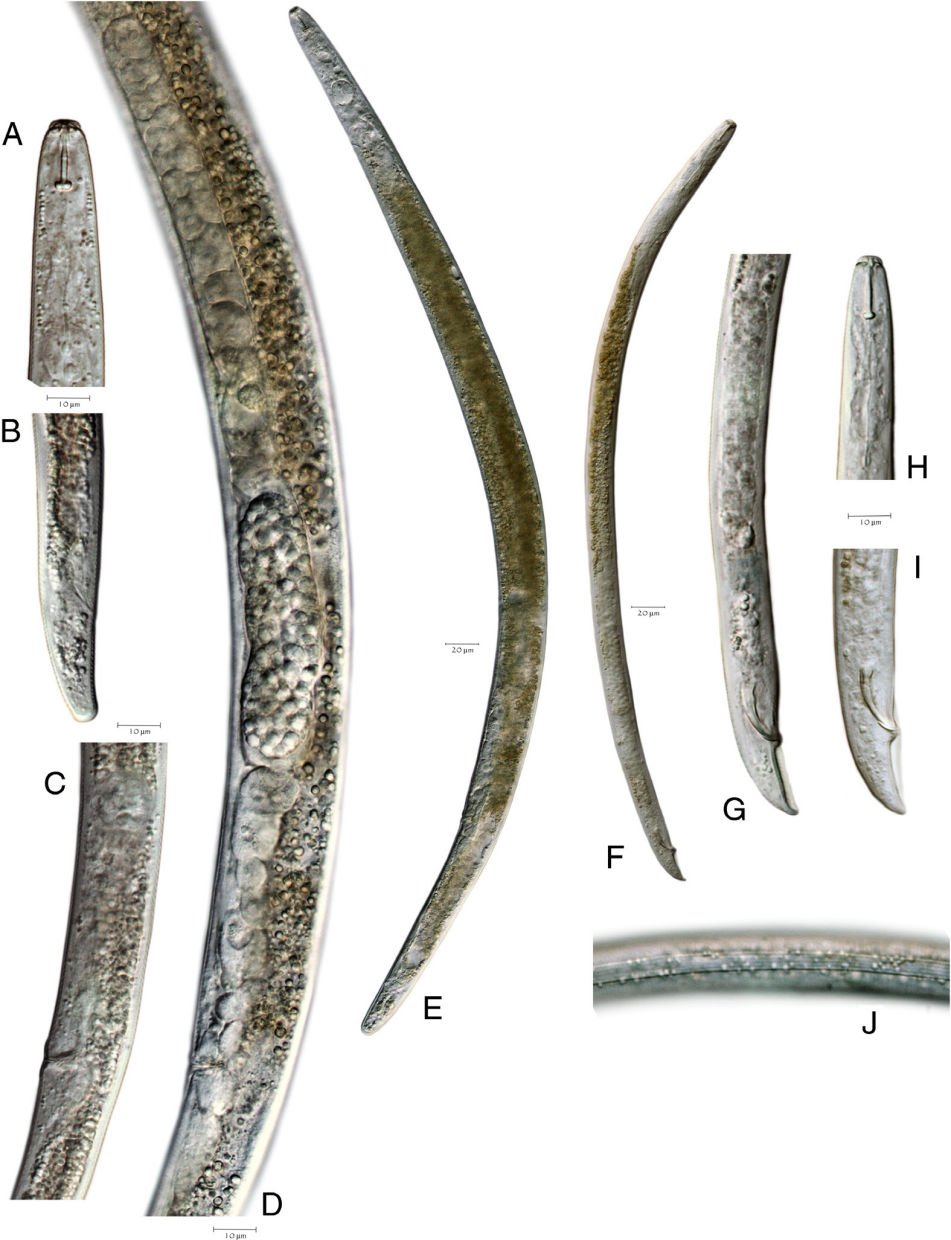
Light microscope images of *Pratylenchus smoliki* n. sp. specimens. (A) NID 8139 female head, (B) NID 8139 female tail, (C) NID 8139 spermatheca, vulva and post-uterine sac, (D) NID 8170 female reproductive system, (E) NID 8170 female entire body, (F) NID 8137 male entire body, (G) NID 8154 spicules and bursa, (H) NID 8154 male head, (I) NID 8137 spicules and bursa, (J) NID 8154 midbody lateral lines in male.

**Figure 2:**
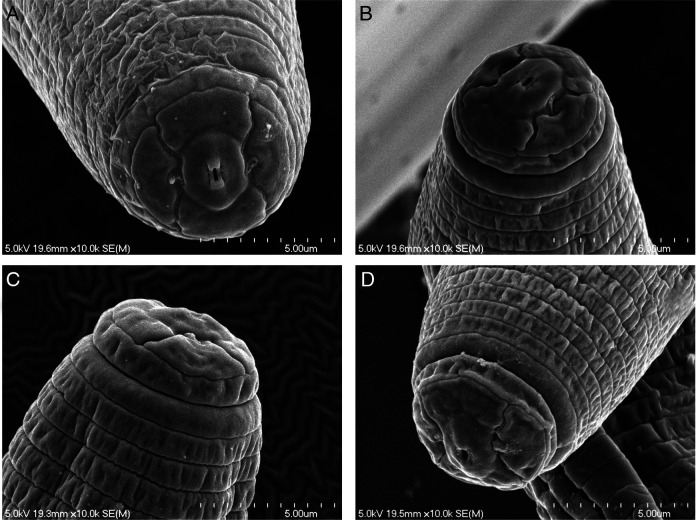
*P. smoliki* n. sp. SEM in *en face* view, different NID numbers specify different individual specimens. (A) NID 4808 Trapezoidal subdorsal and subventral lips adjoining oral disc with demarcation between sectors, six inner labial sensilla flank oral opening, (B) NID 4801 Lateral lip sectors clearly separated from oral disc and subventral/subdorsal sectors, (C) NID 4805 and (D) NID 4820 Anterior region profiles displaying two lip annuli.

**Figure 3:**
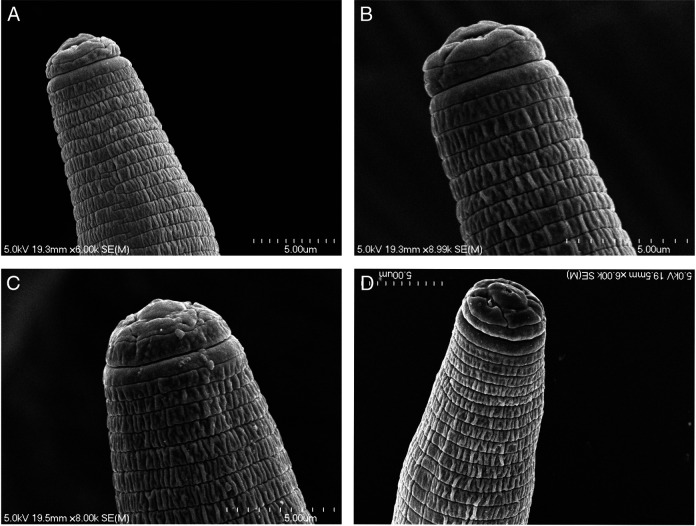
*P. smoliki* n. sp. SEM of anterior region with beginning of lateral field, different NID numbers specify different individual specimens. (A) NID 4815, (B) NID 4812, (C) NID 4816, (D) NID 4817.

**Figure 4:**
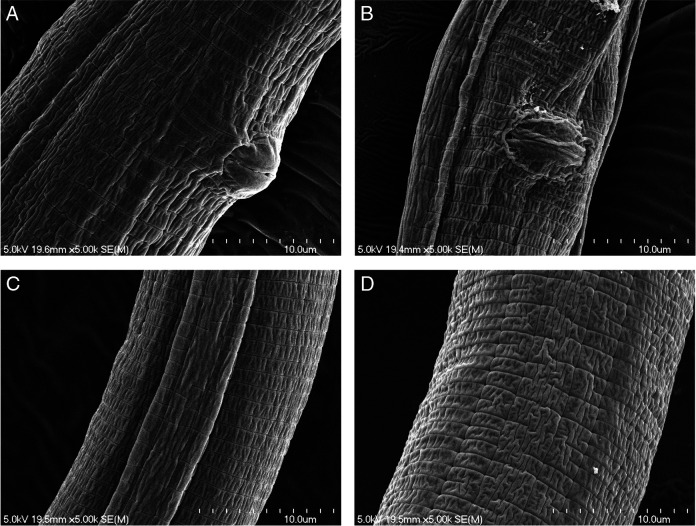
*P. smoliki* n. sp. SEM of midbody region, different NID numbers specify different individual specimens. (A) NID 4808 and (B) NID 4818 slightly protruding vulval lips, (C) NID 4807 and (D) NID 4803 lateral field with irregularly areolated lines at midbody.

**Figure 5:**
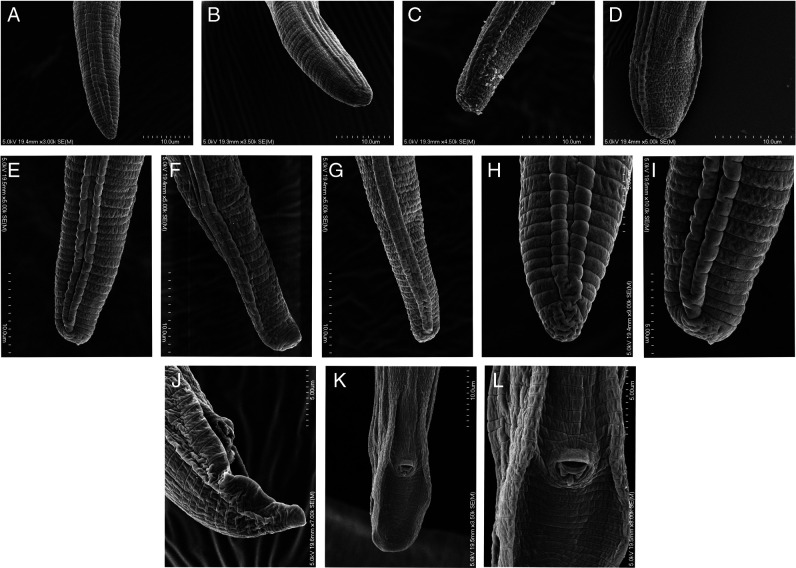
SEM images of *P. smoliki* n. sp. tails, different NID numbers specify different individual specimens. A–I exhibit the variation in tail termini, J–L illustrate male tails. (A) NID 4803, (B) NID 4819, (C) NID 4813, (D) NID 4818, (E) NID 4805, (F) NID 4811, (G) NID 4809, (H) NID 4803, (I) NID 4805, (J) NID 4817, (K) NID 4816, (L) NID 4816.

Pratylenchus smoliki* n. sp. (Figs. 1, 2, 3, 4, 5)

*  The specific epithet is proposed to honor Dr. James Smolik

Figure 1. (Plate of *Pratylenchus smoliki* n. sp. light micrograph)

Figures 2–5. (Plates of SEM *Pratylenchus smoliki* n. sp.—Plate of heads, plate of profiles, plate of mid-body, plate of tails)

Measurements see Table 3. (Table 3. Morphometric parameters of live specimens of *Pratylenchus smoliki* n. sp. from corn, Shawnee County, KS, USA. Measurements are in μm and in the form ±SD (range)).

**Table 3.** Measurements of *P. smoliki* n. sp. specimens.

	Holotype	*N*	Females	*N*	Males	*N*	Juveniles
L	491	22	511.4 + 50.6 (407-604)	25	456.8 + 32.7 (415-560)	4	320.5 + 38.1 (289-385)
a	21.0	22	24.4 + 3.0 (18.8-31.9)	25	27.1 + 1.7 (23.8-31.7)	4	20.5 + 2.3 (18.0-24.1)
b	5.4	21	5.6 + 0.4 (4.8-6.6)	25	5.3 + 0.4 (4.4-5.9)	4	3.7 + 0.5 (3.3-4.5)
b′	4.2	22	4.3 + 0.4 (3.5-4.9)	25	4.1 + 0.3 (3.6-4.6)	4	3.0 + 0.3 (2.5-3.4)
c	19.5	22	22.1 + 2.8 (17.7-31.5)	25	22.6 + 3.9 (16.7-34.1)	4	17.7 + 2.2 (15.7-21.5)
c′	1.9	22	1.9 + 0.2 (1.6-2.2)	24	1.6 + 0.2 (1.2-2.2)	4	1.8 + 0.1 (1.7-1.9)
V %	79.4	22	79.2 + 1.4 (76.8-82.0)				
Lip annules	2	22	2.0 + 0 (2-2)	25	2.0 + 0 (2-2)	4	2.0 + 0 (2-2)
St	16	22	15.1 + 0.6 (14-16)	25	14.2 + 0.6 (13-15)	4	12.7 + 0.6 (12-14)
M %	50	22	48.3 + 1.6 (44.9-51.7)	25	47.7 + 2.9 (43.1-54.7)	4	44.9 + 2.5 (41.9-48.7)
DGO	3	22	2.7 + 0.3 (2-3)	25	2.9 + 0.1 (3-3)	4	2.3 + 0.1 (2-3)
O %	17.3	22	17.7 + 1.9 (12.7-20.3)	25	20.0 + 1.2 (18.3-23.0)	4	18.3 + 1.3 (16.5-20.0)
MB %	45.1	22	44.1 + 2.5 (39.4-51.7)	25	44.6 + 2.6 (39.8-50.1)	4	42.0 + 3.2 (39.2-47.1)
Overlap		22	29.1 + 6.5 (16-41)	25	26.2 + 6.4 (14-39)	4	19.4 + 8.3 (7-27)
Body ann W	1.6	22	1.4 + 0.2 (1.0-2.0)	24	1.3 + 0.1 (1.0-1.5)	4	1.2 + 0.1 (1.0-1.3)
LFL	4			24	4.0 + 0.0 (4-4)		
PUS/Bw	0.6	22	0.9 + 0.2 (1-1)				
PUS/V-A %	16.1	22	22.5 + 6.4 (13.5-32.8)				
Sthc L/W		22	1.1 + 0.1 (0.9-1.4)				
T %				25	44.1 + 4.8 (35.0-55.0)		
Spicule				25	16.8 + 0.8 (15-19)		
Gubernaculum				24	4.3 + 0.6 (3-6)		
Bursa: % tail				25	96.5 + 9.2 (80.5-136.5)		
Tail	25	22	23.4 + 2.8 (17-29)	25	20.7 + 3.2 (13-28)	4	18.2 + 0.4 (18-19)
T/VA	0.3	22	0.3 + 0.0 (0.2-0.4)				
Ran	21	22	19.7 + 3.0 (14-26)	6	18.6 + 3.0 (15-24)	4	18.0 + 1.2 (16-19)

## Description

### Females

Body slender, vermiform, tapering anteriorly, ventrally arcuate to slightly sinuate when relaxed. Body annuli approximately 1 to 2 μm wide at mid-body. Lateral field mostly with four lines, but sometimes supplemented with additional inner lines or striae from midbody to the vulval region, giving the appearance under the light microscope of 5, 6 or even 10 lines. Lateral field beginning 7.1 ± 1.5 (4-11) μm from the anterior end and 7.2 ± 1.6 (5-11) μm wide, occupying 23 to 45% of body diameter at mid-body. Areolation not readily observed in light microscopy, but more apparent in SEM. Lateral field extending 83 to 91% of tail length, terminating 2.6 ± 0.6 (2-4) μm from the tail tip.

Lip region cap-like, narrower than the succeeding body contour, with two lip annuli; 2.3 ± 0.3 (2-3) μm high and 7.3 ± 0.5 (6-8) μm wide, anterior margin truncate with rounded edges. Head immediately posterior to second lip annulus 9.4 ± 0.7 (8-11) μm wide. Cephalic framework moderately developed.

*En face* view characterized by a divided face with rectangular to trapezoidal subdorsal and subventral lips adjoining oral disc, but with a clear demarcation between the oral disc and the subdorsal and subventral sectors. The lateral lip sectors are separated from the disc and subventral/subdorsals by two incisures doubly bent, forming an obtuse angle (pattern “I” sensu Subbotin et al., 2008). This lip pattern configuration is intermediate between groups 2 and 3, according to the classification scheme of Corbett and Clark (1983), differing especially in the separate oral disc.

Stylet short, robust; knobs rounded to rhomboid, flat or slightly indented anteriorly and 4.5 ± 0.4 (4-5) μm wide. Dorsal pharyngeal gland orifice located 2.7 ± 0.3 (2-3) μm posterior to base of knobs. Median bulb muscular, round to ovoid, 13.1 ± 1.3 (11-16) long × 10.7 ± 0.7 (9-12) μm wide, occupying 51-73% of the corresponding body diameter. Cuticularized valve plates prominent. Nerve ring encircling isthmus, 66.7 ± 4.5 (58-75)  μm from anterior end. Isthmus 16.5 ± 3.7 (10-22)  μm long, about 2  μm wide. Hemizonid 3.1 ± 0.3 (2-4)  μm long, located up to 7  μm anterior to secretory-excretory pore, but usually within 2 to 5 μm. Secretory-excretory pore usually anterior to pharyngo-intestinal junction. Pharyngeal glands in tandem, elongate, overlapping intestine ventrally, 43.9 ± 7.5 (28-68) μm long; pharyngeal gland nuclei in tandem. Intestine lacking fasciculi. Reproductive system monodelphic, prodelphic, 169.7 ± 36.1 (91-258) μm long, ovary outstretched with single row of oocytes, vulva usually slightly less than 80% of total body length from anterior end, vulval lips usually slightly protruding, occasionally slightly sunken; lateral flaps and epiptygma absent. Spermatheca rounded to ovoid, 13.9 ± 2.8 (8-19) μm long by 13.1 ± 2.3 (7-18) μm wide, containing spherical sperm, often with distinct nuclei. Post-vulval uterine sac 18.3 ± 4.2 (10-27) μm long, 0.9 times anal body diameter or about 22.5% of the vulva-anus distance. Distance from vulva to anus 82.9 ± 11.9 (63-98) μm. Tail short, conoid to subcylindrical, sometimes slightly ventrally arcuate, with 14 to 26 annuli. Tail tip blunt, subhemispherical to truncate, smooth or slightly irregular. Phasmid pore-like, located 7 to 11 annules posterior to anus, 27 to 51% of total tail length. Hyaline portion of tail terminus 1.8 ± 0.5 (1-3) μm thick.

### Males

Abundant, morphology generally similar to that of female, [including *en face* morphology] and sexual dimorphism. Lip region characters as in female, but more rounded in outline. Some individuals observed with two lip annules on one side (dorsal or ventral) and three on the other. Lateral fields marked by four lines, without apparent areolation when viewed by light microscopy; additional lines or striae not observed. Reproductive system consisting of a single testis anteriorly outstretched, extending 201.4 ± 25.5 (157-273) μm anterior to cloaca. Spicules arcuate, cephalated; gubernaculum slightly curved. Tail conical, ventrally arcuate or bent, sometimes slightly digitate, with finely rounded to bluntly pointed terminus. Bursa 33.5 ± 3.8 (26-40) μm long, varying in extent from 80 to 137% of tail length. Ventral surface of bursa slightly to moderately crenate, occasionally notched. Phasmid pore-like, 9.1 ± 2.1 (4-14) μm posterior to anus, at 31 to 60% of total tail length.

### Type locality and habitat

Holotype Specimen Measurements (nematode identification number [NID] 9793) from the Kansas River Valley (KRV) Experiment Field, near Silver Lake, Kansas.

### Type material

Holotype tissue from NID9793 has been deposited with accession number P-2021-052 and catalog number HWML-112258 in the Harold W. Manter Laboratory of Parasitology, W-529 Nebraska Hall, University of Nebraska State Museum curated by Dr. Scott Gardner. Three additional paratype slides, each containing two females and two males, were distributed to the United States Department of Agriculture Nematode Collection, Beltsville, MD, USA. As required by the International Commission on Zoological Nomenclature, the ZooBank registration number for the new Linnaean binomials is LSID urn:lsid:zoobank.org:pub:9B64E021-C835-420A-A479-F5E688DC61B3.

### Voucher material

Two slides, each containing two females and two males, of the reference *P. smoliki* n. sp. (Silver Lake, Kansas) populations were distributed to each of the following collections: University of California Riverside Nematode Collection, Riverside, USA and University of California Davis Nematode Collection, Davis, USA.

### Differential diagnosis

*Pratylenchus smoliki* n. sp. Is characterized by an offset lip region bearing two annuli which are distinctly narrower than the head region immediately posterior, SEM face divided into clearly-demarcated oral disc, with separate lateral and submedial lip sectors forming an obtuse, doubly bent angle with the labial disc, corresponding to pattern I in Subbotin et al. (2008), stylet 15 (14-16) μm long, with rounded knobs, pharyngeal overlap fairly short (1-2 corresponding body diameters in length), lateral field faintly and occasionally areolated as viewed in light microscopy, and with four incisures plus additional striae, especially at midbody, body annulation moderate, spermatheca round, filled with sperm, vulval lips slightly protruding, tail conoid to subcylindrical with blunt, usually smooth terminus, males common, with slightly smaller stylet (14 [13-15] μm), lateral field with 4 lines, spicules 17 (15-19) μm in length and ventrally curved, bluntly pointed tail.

The matrix code of the new species, according to Castillo and Vovlas (2007) is: A1, B2, C2, D2, E2(3), F2(34), G(2)3, H1, I1(23), J1(23), K1.

The main morphological characters distinguishing *Pratylenchus smoliki* n. sp. include the two lip annuli, functional, rounded spermatheca, stylet about 15 μm in length, more posterior vulva (V > 75%), conoid to subcylindrical tail with smooth terminus, and abundant males. Similar *Pratylenchus* species, based on this combination of characters, along with the results of our phylogenetic analyses, include *P. alleni* Ferris, 1961, *P. hexincisus* Taylor & Jenkins, 1957, *P. pseudocoffeae* Mizukubo, 1992, and *P. scribneri* Steiner in Sherbakoff & Stanley, 1943. The latter three species all have basically the same face pattern (type I in Subbotin et al., 2008) as *P. smoliki* n. sp. when viewed with SEM. The face pattern of *P. alleni* has not been clearly established, but preliminary results of our SEM studies indicate a similar general appearance.

**Figure 6:**
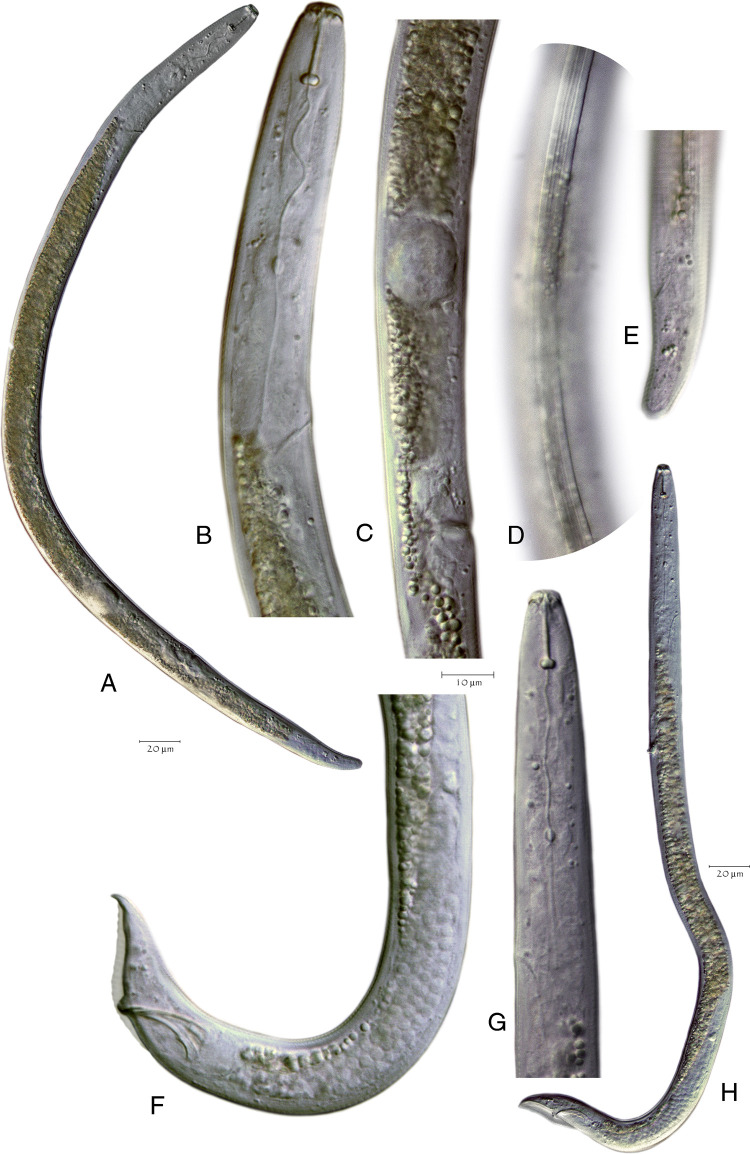
Light microscope images of *Pratylenchus alleni* specimens from the type locality Saline County, Illinois. (A–E) female NID 7381, (F–H) male NID 7382. (A) Entire body, (B) Head and anterior region, (C) Reproductive region, (D) Midbody lateral lines, (E) Tail, (F) Tail, bursa and spicules, (G) Head and anterior portion, (H) Entire body

*Pratylenchus smoliki* n. sp. differs from *P. alleni* mainly by the longer body (usually > 500 μm vs maximum 440 μm), and in the shorter pharyngeal overlap (< 30 μm vs >40 μm), along with generally shorter post-vulval uterine sac (PUS), relative to vulval body width (PUS/vbw; 0.9 vs 1.1) and slightly more anterior vulva (<80% vs >80%), although the value ranges for these characters overlap (Figure 6).

From *P. hexincisus,* the new species differs in having numerous males (vs rare) and a functional, rounded spermatheca (vs. spermatheca absent or reduced), along with greater body length and c ratio, and lower b and c’ values (mean values: 511 μm vs 453 μm, 22.1 vs 18.4, 5.6 vs 6.1, and 1.9 vs 2.3, respectively). *P*. *smoliki* n. sp. also usually displays fewer lateral field lines in the advulval region (4-6 vs 6-8).

*Pratylenchus smoliki* n. sp. can be differentiated from *P. pseudocoffeae* based on spermatheca shape (round vs oval), and shorter PUS (<20 μm vs >25 μm). Overall, *P. smoliki* n. sp. has lower values for a, c’, V and stylet length (mean values: 24.4 vs 27.6, 1.9 vs 2.2, 79.2 vs 81.0, and 15.1 vs 16.0 μm, respectively), and a higher mean c value (22.1 vs 19.3).

*Pratylenchus smoliki* n. sp. differs from *P. scribneri* in spermatheca shape (round vs oval), shorter PUS (<20 μm, PUS/vbw 0.9 vs >25 μm, PUS/vbw 1.3), shorter pharyngeal overlap (< 30 vs > 30 μm), lower mean a value (< 25 vs > 25) and a generally shorter tail (mean *c* 22.1 vs 17.5, mean *c*′ 1.9 vs 2.4).

It should be noted, however, that overlap of the range values of all of these morphological characters makes the separation of these species by morphology alone difficult and unreliable.

Other *Pratylenchus* species with different SEM face patterns, but sharing with *P. smoliki* n. sp. the combination of two lip annuli, functional spermatheca, presence of males, and lateral field non-areolated with four lines at vulval region include *P. coffeae* (Zimmermann, 1898) Filipjev & Schuurmans Stekhoven, 1941, *P. flakkensis* Seinhorst, 1968, *P. loosi* Loof, 1960, and *P. silvaticus* Brzeski, 1998.

*P. smoliki* n. sp. can be differentiated from *P. coffeae* by its shorter stylet (mean stylet length 15.1 vs 16.3 μm) and pharyngeal overlap (< 30 vs > 30 μm), round (vs oval) spermatheca, and shorter PUS (<20 vs >35 μm). Overall, *P. smoliki* n. sp. has lower values for *L*, *a*, *b*, *c*′ and V (mean values 511, 24.4, 5.6, 1.9, 79.2 vs 578, 28.6, 6.4, 2.3, 80.3, respectively). The face pattern for *P. coffeae* shows a mostly set off labial disc, but no clear separation of the lateral or submedial lips and thus no angle formed between the laterals and the labial disc (pattern J of Subbotin et al., 2008).

From *P. flakkensis, P. smoliki* n. sp. differs in its shorter tail (mean *c* 22.1 vs 17.2, mean *c*′ 1.9 vs 2.5) with smooth (vs annulated) terminus and shorter stylet (< 16 vs > 16 μm). In *P. flakkensis*, the lateral and submedial lips are not fused and the laterals form an obtuse, doubly-bent angle, similar to the pattern in *P. smoliki* n. sp, but the labial disc is not set off (pattern H in Subbotin et al., 2008).

Compared with *P. loosi*, the new species differs in having a rounded (vs rectangular) spermatheca, more anterior vulva (mean V value 79.2% vs 82.0%), smooth and blunt tail terminus (vs pointed) and shorter stylet (average length 15.1 vs 16.0 μm). In general, *P. smoliki* n. sp. is shorter (mean L 511 vs 588 μm), stouter (mean a 24.4 vs 29.4), with a shorter tail (mean c 22.1 vs 19.3, mean c’ 1.9 vs 2.8) and slightly lower b ratio (mean value 5.6 vs 6.4). The face pattern for *P. loosi* is similar to that of *P. coffeae* (above).

*P. smoliki* n. sp. differs from *P. silvaticus* by its shorter pharyngeal overlap (<30 vs >30 μm), longer body (mean L 511 vs 450 μm), more posterior vulva (mean *V* <80% vs >80%), and shorter tail (mean c’ 1.9 vs. 2.4). In addition, average values for b (5.6 vs 6.3) and PUS relative to vulval body width (0.9 vs 1.3) are lower in *P. smoliki* n. sp. No *en face* pattern data for *P. silvaticus* are available.

Also morphologically similar is *P. neglectus* (Rensch, 1924) Filipjev & Schuurmans Stekhoven, 1941, which generally lacks males, except in rare instances. *P. smoliki* n. sp. females have a prominent, functional spermatheca (vs. absent or reduced in *P. neglectus*), longer PUS (> 16 vs < 16 μm), more anterior vulva (mean V of 79.2% vs 82.0%), shorter stylet (mean stylet length 15.1 vs 16.5 μm), and shorter tail (mean c value 22.1 vs 19.8, mean *c*′ 1.9 vs 2.2). In *en face* view, the labial disc of *P. neglectus* is not offset, and forms a simple (single inflection), obtuse angle with the lateral lip sectors.

Recently another new species of *Pratylenchus*—*P. dakotaensis* Handoo, Yan, Kantor, Chowdhury, Plaisance, Bauchan & Mowery, 2021—from North Dakota (USA) has been described. DNA sequence data readily differentiate *P. dakotaensis* from *P. smoliki* n. sp., despite the biome similarity of Great Plains type localities. In addition, *P. smoliki* n. sp. differs from *P*. *dakotaensis* in number of lip annules (2 vs 3), tail terminus morphology (smooth under LM vs crenate), and lateral field characteristics (outer bands generally plain under LM in *P. smoliki* n. sp. vs areolated in *P. dakotaensis*). The two species also differ in *en face* pattern: as discussed above, *P. smoliki* n. sp. displays a pattern similar to type I of Subbotin et al. (2008), with a more set-off labial disc, while the face of *P. dakotaensis* conform to type H, similar to that of *P. flakkensis*, with the labial disc not demarcated.

**Figure 7:**
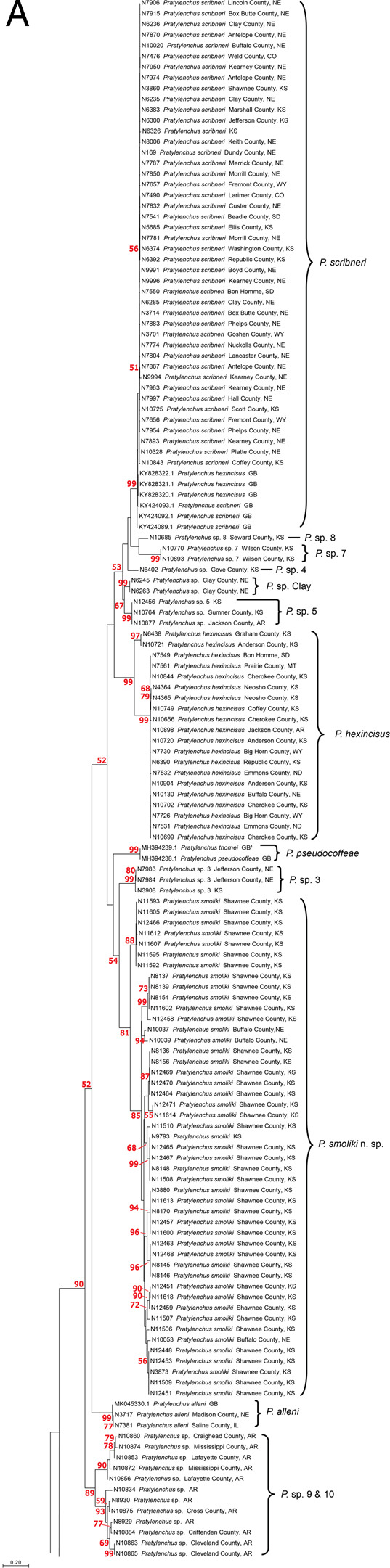

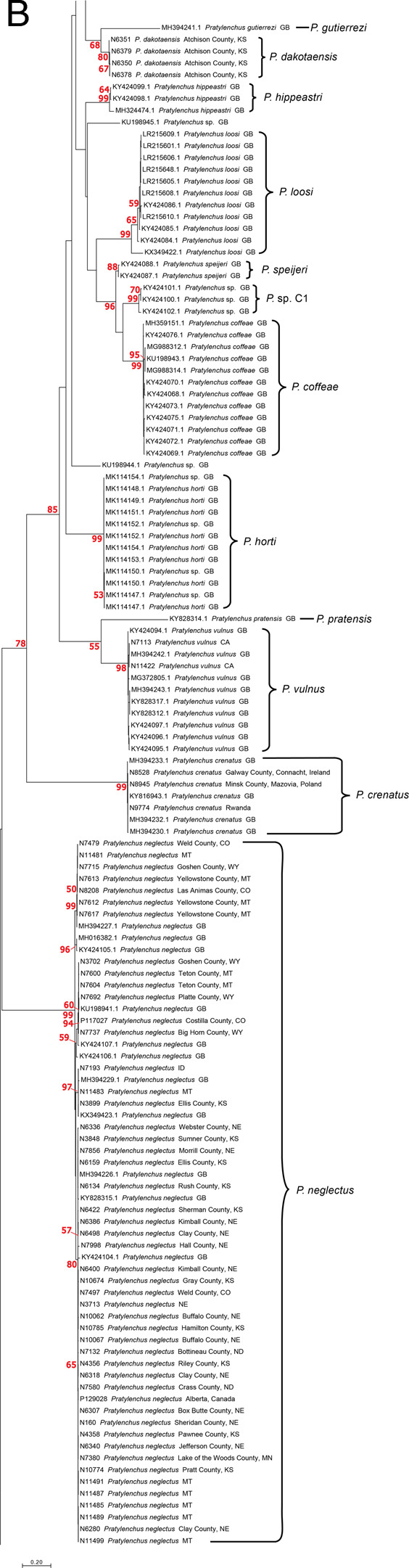

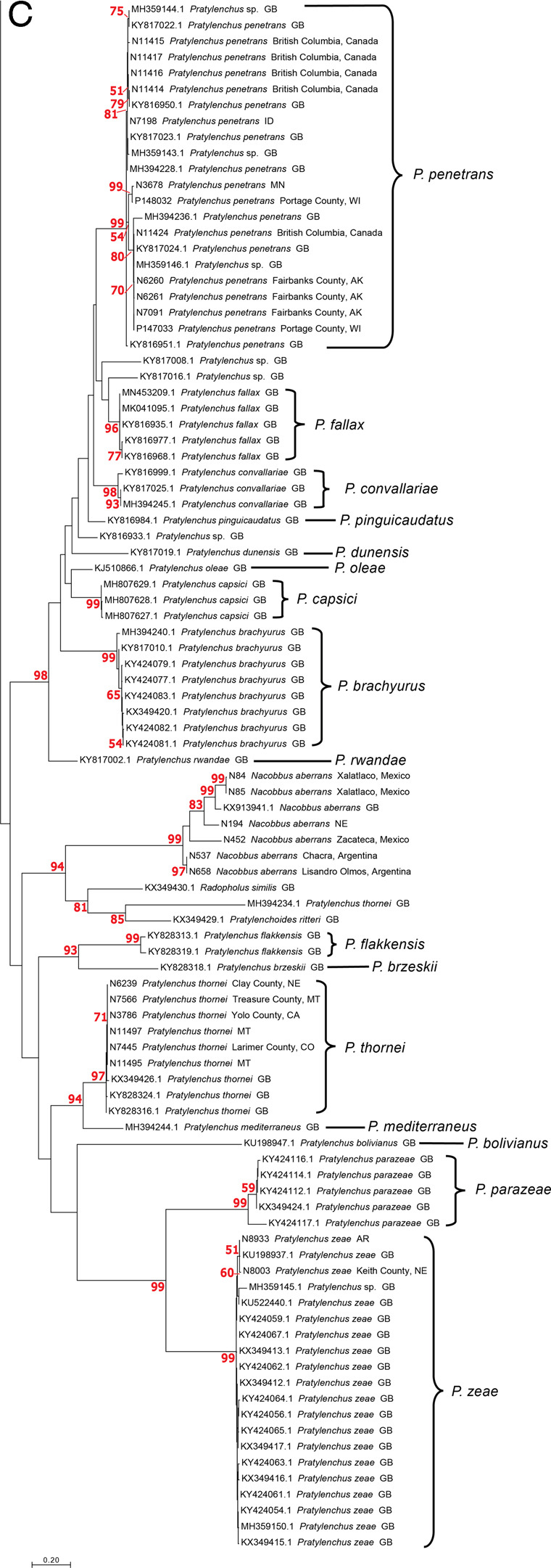
A-C, COI DNA Maximum likelihood tree of 372 Pratylenchid sequences GTR + G 200, the tree is split into three sections, 7A, 7B, and 7C. Bootstrap values in red, brackets delimit species and haplotype groups.

**Figure 8:**
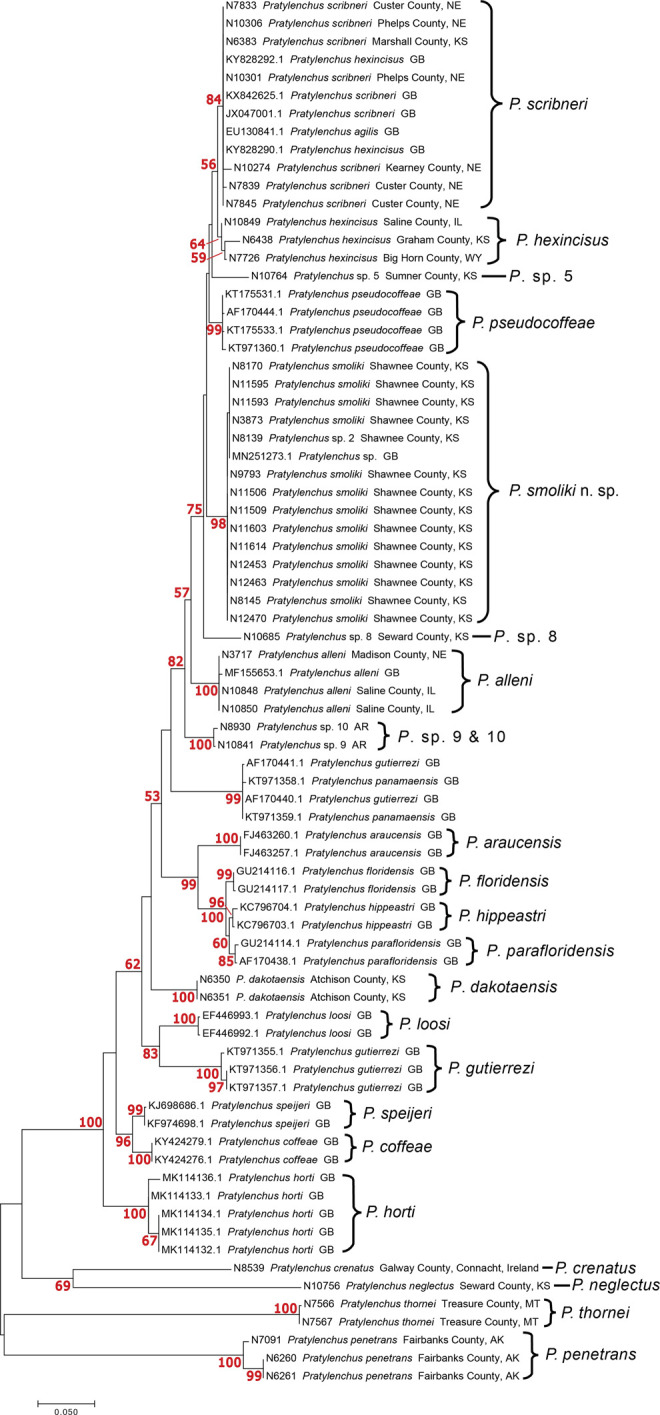
28S DNA Maximum likelihood tree of 41 Pratylenchid sequences K2P + G 500 Bootstrap values in red, brackets delimit species and haplotype groups.

**Figure S1:**
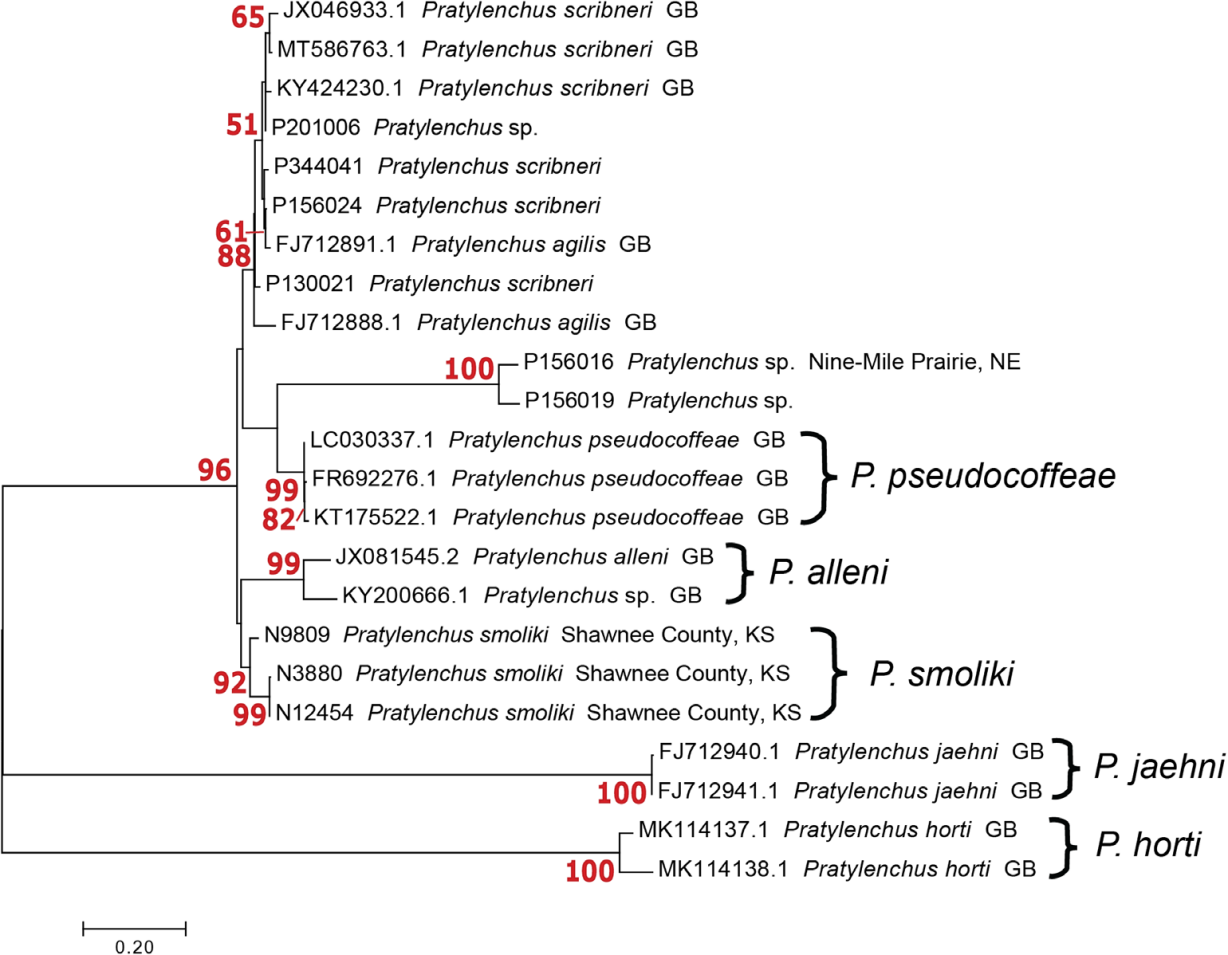
Maximum likelihood tree of ITS1 sequences from *P. smoliki* n. sp. and closely related species. Bootstrap values in red.

*Phylogeny:* The maximum likelihood COI tree of *Pratylenchus* species includes 47 specimens from Kansas and Nebraska that belong to a clade representing *Pratylenchus smoliki* n. sp (Figs. 6 and 7). The clade is supported by a bootstrap support value of 81, with subgroups within the clade that indicate differentiation at a subspecific level. The *P. smoliki* n. sp. clade includes specimens of all life stages. Thirteen of the *P. smoliki* n. sp. specimens on the COI tree are also represented on 28S ML tree (Fig. 8). A bootstrap support value of 98 characterizes the *P. smoliki* n. sp. clade on the 28S tree, and a GenBank sequence from Wisconsin suggests that the geographic range of the species may extend to three U.S. states. The 28S tree has support at deeper nodes in the tree and allows recognition of *P. smoliki* n. sp. as a member of a group consisting of four related described species (*P. scribneri*, *P. hexincisus*, *P. ps*e*udocoffeae, P. alleni*) and a putative undescribed species labeled as haplotype groups 9 and 10 in Figure 7. This grouping of species is consistently supported by 28S, 18S, and ITS trees in the literature (Inserra et al., 2007; Subbotin et al., 2008; Araya et al., 2016; Troccoli et al., 2016; Janssen et al., 2017; Singh et al., 2018; Nguyen et al., 2019; Ozbayrak et al., 2019; Handoo et al., 2021) and is highly congruent with grouping based on lip patterns. A maximum likelihood ITS1 tree also illustrates the distinctiveness of *P. smoliki* n. sp. (Supplementary Fig. S1). Although higher evolutionary rates in the COI gene constrains its use for deeper divergences on the tree, it is often the case that COI haplotype groups of described species are consistent with species boundaries established by other morphological and molecular characters. However, within the genus *Pratylenchus* a considerable amount of mitochondrial genetic differentiation has occurred, and for some species such as *P. smoliki* n. sp., the population-level variation is relatively high (Table 4; Ozbayrak et al., 2019 Table 3). Low amounts of haplotype diversity and a simple population structure may reflect recent dispersal characteristic of an introduced species (Clavero et al., 2016). *P. thornei* is a good example of a potentially introduced species to North America, where a single haplotype is spread across six western states in the U.S. (Ozbayark et al., 2019). Conversely, multiple genetic subgroups may reflect a native origin with a higher level of differentiation among regional populations (Grenier et al., 2010, Powers et al., 2016). *Pratylenchus* isolates from native plant communities will help resolve the issue of natives versus introduced status for *Pratylenchus* species.

**Table 4.** Estimates of evolutionary divergence (p-distances) over sequence pairs within (bold) and between groups.

	N. aberrans	P. alleni	P. crenatus	P. hexicisus	P. neglectus	P. penetrans	P. scribneri	P. sp. 3	P. sp Clay	P. dakotaensis	P. smoliki n. sp.	P. sp. 9 & 10	P. sp. 7	P. sp. 5	P. thornei	P. vulnus	P. zeae
Nacobbus aberrans	0.105																
P. alleni	0.328	0.003															
P. crenatus	0.358	0.316	0.001														
P. hexincisus	0.416	0.217	0.36	0.029													
P. neglectus	0.314	0.3	0.36	0.381	0.02												
P. penetrans	0.325	0.323	0.32	0.379	0.31	0.032											
P. scribneri	0.36	0.172	0.32	0.198	0.329	0.334	0.007										
P. sp. 3	0.365	0.181	0.356	0.23	0.339	0.348	0.187	0.004									
P._sp_Clay	0.373	0.188	0.329	0.196	0.329	0.339	0.112	0.191	0								
P. dakotaensis	0.335	0.216	0.306	0.286	0.322	0.307	0.242	0.228	0.247	0.078							
P. smoliki n. sp.	0.412	0.219	0.381	0.21	0.377	0.362	0.227	0.19	0.221	0.298	0.101						
P. sp. 9 & 10	0.378	0.204	0.336	0.23	0.355	0.35	0.21	0.226	0.205	0.254	0.229	0.105					
P. sp. 7	0.351	0.171	0.303	0.215	0.311	0.321	0.142	0.188	0.15	0.212	0.235	0.212	0.091				
P. sp. 5	0.38	0.195	0.333	0.179	0.358	0.349	0.127	0.2	0.079	0.26	0.206	0.204	0.161	0			
P. thornei	0.311	0.34	0.372	0.386	0.298	0.311	0.355	0.352	0.348	0.32	0.373	0.349	0.328	0.366	0.002		
P. vulnus	0.335	0.241	0.321	0.3	0.333	0.305	0.265	0.287	0.276	0.241	0.319	0.259	0.246	0.297	0.316	0.051	
P. zeae	0.326	0.326	0.346	0.398	0.315	0.331	0.352	0.332	0.34	0.314	0.394	0.36	0.331	0.36	0.317	0.327	0.012
P. horti	0.331	0.26	0.331	0.296	0.317	0.31	0.243	0.23	0.244	0.217	0.308	0.246	0.228	0.277	0.295	0.254	0.299
P. fallax	0.279	0.302	0.292	0.359	0.278	0.153	0.311	0.332	0.321	0.271	0.39	0.342	0.297	0.346	0.272	0.296	0.309
P. pseudocoffeae	0.326	0.18	0.339	0.22	0.326	0.326	0.193	0.181	0.173	0.243	0.229	0.222	0.184	0.197	0.345	0.277	0.331
P. coffeae	0.314	0.231	0.28	0.302	0.324	0.293	0.229	0.249	0.237	0.224	0.291	0.24	0.224	0.257	0.304	0.244	0.303
P. hippeastri	0.307	0.234	0.318	0.3	0.325	0.307	0.247	0.246	0.255	0.201	0.278	0.256	0.228	0.267	0.326	0.246	0.317
P. loosi	0.301	0.223	0.311	0.302	0.281	0.302	0.242	0.236	0.246	0.215	0.301	0.248	0.235	0.263	0.298	0.271	0.296
P. convallariae	0.315	0.337	0.316	0.385	0.316	0.167	0.329	0.349	0.333	0.291	0.389	0.346	0.308	0.351	0.31	0.305	0.323
P. sp	0.324	0.222	0.287	0.284	0.309	0.309	0.228	0.242	0.254	0.204	0.288	0.242	0.227	0.252	0.32	0.25	0.296
P. speijeri	0.306	0.235	0.29	0.274	0.307	0.312	0.216	0.253	0.233	0.198	0.3	0.236	0.224	0.248	0.305	0.231	0.306
P. capsici	0.299	0.313	0.329	0.365	0.296	0.194	0.312	0.319	0.317	0.277	0.368	0.342	0.306	0.339	0.296	0.3	0.326
P. brachyurus	0.316	0.323	0.339	0.396	0.31	0.233	0.341	0.341	0.366	0.296	0.388	0.371	0.328	0.369	0.283	0.317	0.328
P. flakkensis	0.317	0.342	0.377	0.392	0.352	0.305	0.356	0.349	0.343	0.324	0.384	0.354	0.336	0.371	0.292	0.335	0.332
P. parazeae	0.386	0.368	0.394	0.398	0.366	0.385	0.368	0.355	0.368	0.353	0.398	0.388	0.359	0.374	0.346	0.359	0.264
	P. horti	P. fallax	P. pseudocoffeae	P. coffeae	P. hippeastri	P. loosi	P. convallariae	P. sp. C1	P. speijeri	P. capsici	P. brachyurus	P. flakkensis	P. parazeae				
P. horti	0.001																
P. fallax	0.292	0.007															
P. pseudocoffeae	0.248	0.325	0														
P. coffeae	0.229	0.291	0.252	0.008													
P. hippeastri	0.224	0.304	0.256	0.185	0.023												
P. loosi	0.221	0.305	0.249	0.193	0.209	0.026											
P. convallariae	0.305	0.145	0.331	0.279	0.297	0.306	0.021										
P. sp. C1	0.238	0.298	0.27	0.143	0.197	0.193	0.296	0.018									
P. speijeri	0.215	0.294	0.241	0.118	0.164	0.181	0.283	0.109	0.005								
P. capsici	0.276	0.169	0.334	0.295	0.278	0.284	0.187	0.292	0.28	0.006							
P. brachyurus	0.308	0.202	0.356	0.308	0.316	0.296	0.229	0.298	0.279	0.211	0.014						
P. flakkensis	0.314	0.292	0.356	0.299	0.291	0.292	0.337	0.335	0.333	0.289	0.315	0.04					
P. parazeae	0.352	0.377	0.389	0.361	0.373	0.37	0.389	0.353	0.357	0.357	0.353	0.356	0.049				

Topotype specimens of *P. alleni* from Saline County, Illinois grouped with *P. alleni* specimens collected from Wisconsin and Nebraska in both COI and 28S trees. This species does not occur frequently in Great Plains corn/soybean rotations compared to the occurrence of *P. scribneri* and *P. neglectus* in these cropping systems. *P. alleni* does appear very similar to *P. smoliki* n. sp. and *P. pseudocoffeae* in SEM face, body, and tail views (Fig. 6; Deimi et al., 2009; Araya et al., 2016) supporting their relatively close genetic relationships.

### Host range trials

**Figure 9:**
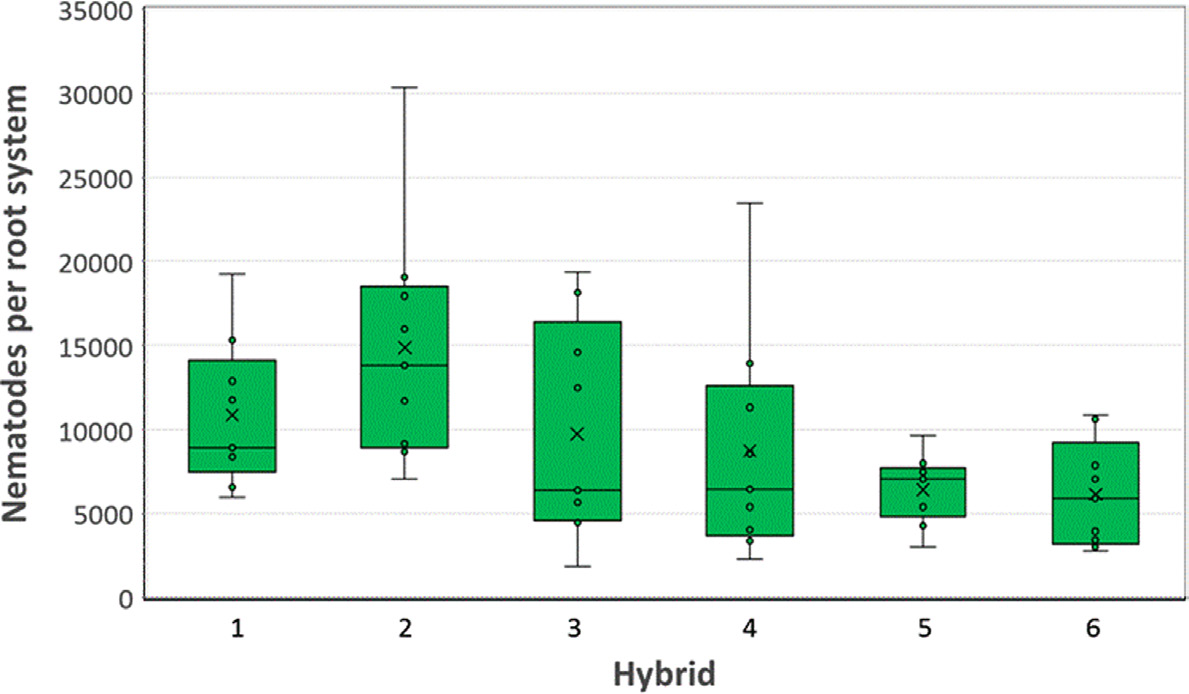
Box and whisker plots of the distribution of numbers of *Pratylenchus smoliki* n. sp. recovered from roots of six eight-week old corn hybrids. The lower bound of the box indicates the first quartile, the central line is the median, and the upper bound is the third quartile. The lower and upper whiskers represent minimum and maximum values. Outliers that are more than 1.5 times the interquartile range (third quartile minus first quartile) from the lower or upper quartile, respectively, are indicated with dots. The mean is indicated by the symbol “×”.

**Figure 10:**
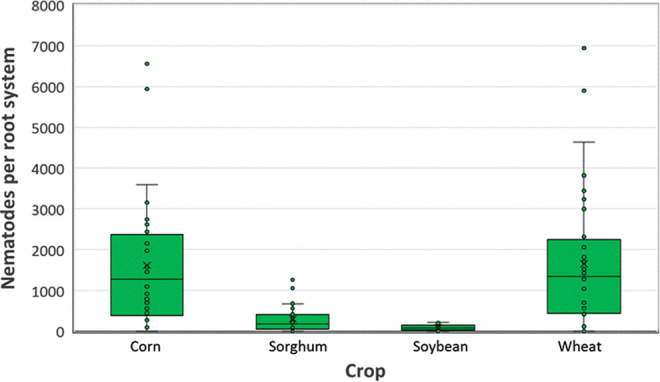
Box and whisker plots of the distribution of numbers of *Pratylenchus smoliki* n. sp. recovered from roots of four eight-week old agronomic crop plants. The lower bound of the box indicates the first quartile, the central line is the median, and the upper bound is the third quartile. The lower and upper whiskers represent minimum and maximum values. Outliers that are more than 1.5 times the interquartile range (third quartile minus first quartile) from the lower or upper quartile, respectively, are indicated with dots. The mean is indicated by the symbol “×”.

**Figure 11:**
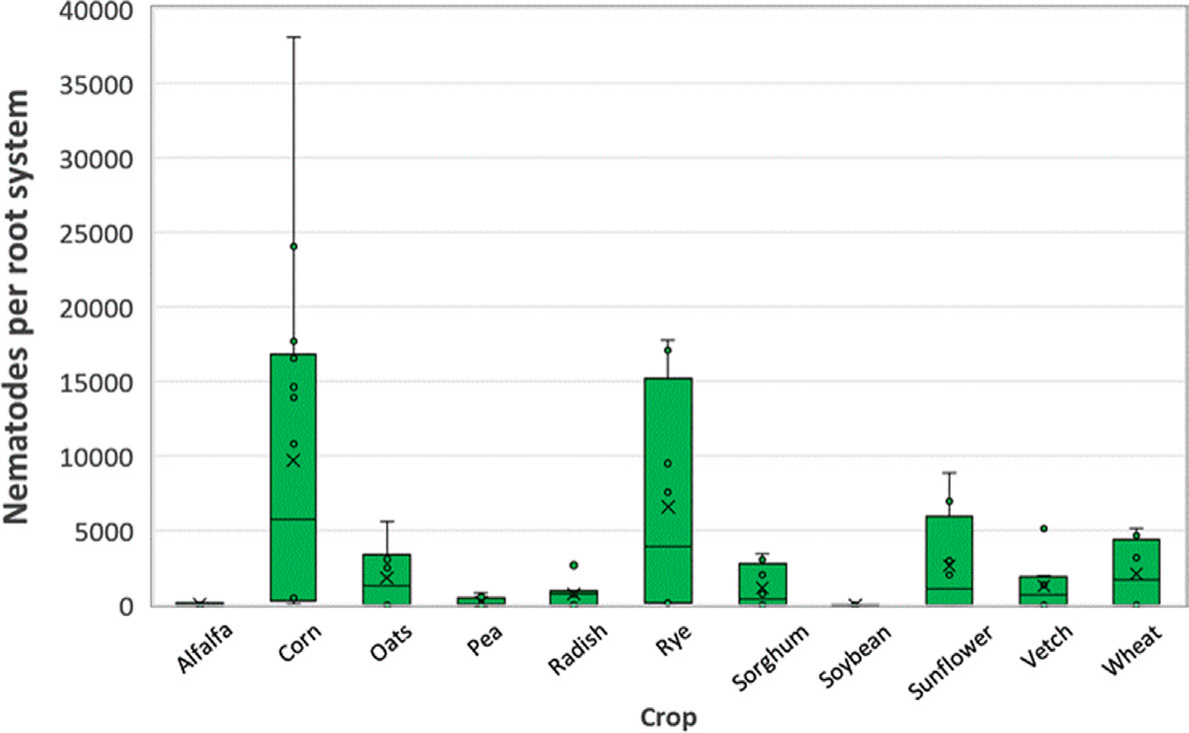
Box and whisker plots of the distribution of numbers of *Pratylenchus smoliki* n. sp. recovered from roots of 11 eight-week old cover crops. The lower bound of the box indicates the first quartile, the central line is the median, and the upper bound is the third quartile. The lower and upper whiskers represent minimum and maximum values. Outliers that are more than 1.5 times the interquartile range (third quartile minus first quartile) from the lower or upper quartile, respectively, are indicated with dots. The mean is indicated by the symbol “×”.

Host range trials were conducted to assess the reproductive potential of *P. smoliki* n. sp. Host status varied with corn hybrid (*p* < 0.001), agronomic crop (*p* = 0.016), and cover crop (*p* < 0.0001). Mean numbers of nematodes recovered from corn roots in corn hybrid trials ranged from 6,169 per pot to 14,864 per pot, with a standard error of 1,782 (Fig. 9). In agronomic crop trials, the largest populations were recovered from corn and wheat, with populations recovered from sorghum and soybean averaging less than 20% of the mean number of nematodes recovered from corn roots (Fig. 10). There was no evidence of a cultivar within crop effect (*p* = 0.98). In cover crop trials, the largest populations were recovered from corn and rye, followed by sunflower and wheat, with less than 30% of the mean number of nematodes recovered from corn roots (Fig. 11). Alfalfa, pea, and radish were associated with the lowest recovered nematode populations, averaging less than 10% of the mean number of nematodes recovered from corn roots.

Root-lesion nematodes are typically characterized as “polyphagous”, with wide host ranges (Castillo and Vovlas, 2007). Nevertheless, large ranges in host suitability have been reported for *Pratylenchus* species, including *P. penetrans* (Belair et al., 2007), and *P. neglectus* and *P. thornei* (Taylor et al., 2000; Vanstone and Russ, 2001; Smiley et al., 2014). While *P*. *smoliki* n. sp. was recovered from the two-month-old roots of every crop plant investigated in the present study, there was a similarly large range in host suitability among those crops, with important implications for nematode management. Corn appears to be the primary agronomic host for *P. smoliki* n. sp., although it should be noted that corn hybrids exhibited a continuum in host suitability. Wheat, rye, and sunflower are also suitable hosts based on the observed nematode population increases, while alfalfa, sorghum, and soybean were consistently associated with little or no nematode population increase. These latter agronomic crops could be recommended as rotational crops for managing this nematode in corn. Additionally, the host status of cover crops (as well as weeds) needs to be considered, as several of these have been reported to be good hosts for agronomically important species of *Pratylenchus* (Miller, 1978; Belair et al., 2007). At least one cover crop (rye) was identified as a risk in terms of host suitability in the present study. Finally, although phylogenetic signals in pathogen host ranges have been suggested to be predictive of novel host associations (Gilbert and Parker, 2016), this signal does not appear to be robust in *P*. *smoliki* n. sp., with the possible exception of the Triticeae. The observed host range might alternatively be explained by ecological fitting (Agosta, 2006), where each of the most suitable crop host species are members of plant families common to the tallgrass prairie: Andropogoneae (e.g. *Andropogon gerardi*), Triticeae (e.g. *Elymus canadensis*), and Asteraceae (e.g. *Helianthus petiolaris*). If this is the case, it suggests that *P*. *smoliki* n. sp. is native to the eastern Great Plains region. The COI phylogeny provides moderate support for this suggestion. The data reported here provide a preliminary record of the host range of *P*. *smoliki* n. sp., however, further research is needed to ascertain the existence of (1) intraspecific variation in host range among *P*. *smoliki* n. sp. populations and (2) varietal differences in host suitability among crop species.
